# Editorial: Cutting edge in systemic lupus erythematosus

**DOI:** 10.1007/s12026-017-8910-6

**Published:** 2017-03-16

**Authors:** David P. D’Cruz, Annegret Kuhn

**Affiliations:** 1 Louise Coote Lupus Unit, Guys and St Thomas’ Hospitals, London, UK; 2 Interdisciplinary Center for Clinical Trials (IZKS), University Medical Center Mainz, Mainz, Germany; 3 Division of Immunogenetics, Tumor Immunology Program, German Cancer Research Center (DKFZ), Heidelberg, Germany

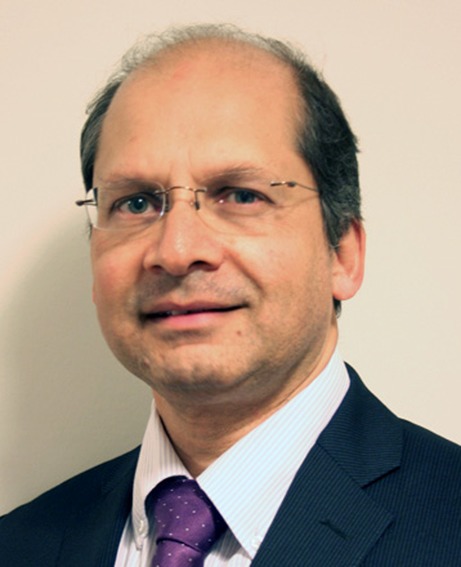



## Editorial

The last 10 years have seen major advances in the understanding of the pathobiology of systemic lupus erythematosus (SLE). These improvements have led to discoveries of new molecules that target specific points in the immunological cascade that contribute to the immune hyperactivity and loss of tolerance in this heterogeneous autoimmune disease. Despite a large number of clinical trials assessing new molecules in patients with active SLE, belimumab (Benlysta®) is the only biologic agent that has been approved for clinical use. Whilst the track record of these large and expensive clinical trials has been disappointing, it has been very encouraging to see that investigators from industry and academia have not been dissuaded from further investment to fill the huge unmet need for better understanding of the immunopathology of SLE and the search for novel and effective treatments.

This special issue of Immunologic Research brings together internationally acclaimed experts in the field of SLE to provide cutting edge updates in this rapidly moving research area We included original articles and reviews to cover basic science and clinical aspects of SLE including the possible role of infections and updates on the antiphospholipid syndrome (APS).

The first original article by Schmidt et al. [1] investigated regulatory T cells (Tregs) in the peripheral blood and inflamed tissue including the skin and kidney in patients with SLE. A reasonable hypothesis is that autoimmune diseases are characterised by overexpression of helper T cells and reduced numbers and/or function of Tregs. However, the literature is very conflicting. Schmidt et al. showed that activated effector T cell populations were increased in the peripheral blood of patients with SLE but that total numbers of Treg cells were unchanged. The numbers of regulatory T cells were similar in the inflamed tissue to those from control diseases. However, specific Treg subsets appeared misbalanced in SLE patients: within CD4^+^CD25^++^ Tregs of patients with active SLE, markers related to Treg function (CD27) and homing (CCR6) were altered. This is a controversial area with conflicting findings in the literature; therefore, this study suggests that analysing subpopulations of Tregs may be more fruitful in dissecting the potential role of these T cells in the pathogenesis of SLE.

Hormones are clearly important in the pathogenesis of SLE and, given that the disorder is 9–10 times more common in females, oestrogens must play a major role. Another hormone that is emerging is prolactin, produced by the anterior pituitary gland and other cells including immune cells. Jara et al. [2] review the potential role of prolactin in the innate and adaptive immune responses in immune-neuroendocrine reactions. Hyperprolactinemia has been demonstrated in association with active disease in SLE. Bromocriptine, a dopamine analogue that suppresses prolactin secretion, may be able to reduce disease activity and improve immune competency in animal models of lupus. However, this mode of treatment has not been widely adopted in patients with SLE.

SLE is commonly a disorder of young women of child-bearing age. Many large lupus centres have clinical services designed to counsel women and manage their pregnancies and the perinatal period. Martinez-Sanchez et al. [3] describe a prospective cohort study of 42 anti-Ro/SSA antibody-positive women and report their pregnancy complications (CHB, preeclampsia, preterm delivery), ultrasound data and modes of delivery. These data complement the previously published information in the literature and will be useful to clinicians tasked with managing these women in their clinics.

Cardiovascular disease is over-represented in women with SLE and whilst the risks are considerably increased, the event rate of cardiovascular disease in SLE is relatively low, making this a difficult area to study when it comes to prevention trials. Szabo et al. [4] summarise the epidemiology of dyslipidemia in SLE patients and review the latest results in the pathogenesis of lipid abnormalities and the assessment and management of dyslipidemia in SLE. For example, patients with lupus nephritis are often characterised by dyslipidemia and it is well recognised that even low-grade proteinuria and renal impairment significantly increase cardiovascular risks.

Watad el al [5] follow this article with analysis of a large health service database in Israel which included over 5000 patients. They demonstrated in a multivariate analysis that SLE was associated with ischemic heart disease with an odds ratio of 3.77.

Vitamin D levels are low in most, if not all, patients with SLE for a variety of reasons. Many patients are photosensitive and therefore have rigorous photoprotection strategies. Marinho et al. [6] argue persuasively that vitamin D not only has a role in the pathobiology of SLE but also that vitamin D treatment is safe and may be effective in modulation of the immune response in active disease. Vitamin D may also influence cardiovascular risk in SLE.

The central role of the microbiome in the maintenance of health and its potential role in a variety of human diseases including autoimmune rheumatic diseases is just beginning to be explored. Katz-Agranov and Zandman-Goddard [7] provide an excellent review of recent studies describing how alterations of the gut microbial composition may be correlated with SLE disease manifestations. These are early days in this exciting field and understanding the gut microbiota may potentially lead to exciting new therapies, perhaps using dietary manipulations to treat lupus.

The possible role of viral infections in the pathogenesis of SLE has long fascinated researchers especially given the central importance of the interferon signature in this disease. However, conclusive evidence has still not been demonstrated in the literature. Hod et al. [8] review the evidence for parvovirus infection in SLE and found that this was very uncommon in their patients (only 3.9% of patients). However, there appeared to be an association between parvovirus infection and antiphospholipid antibodies and these tantalising data will hopefully lead to confirmatory studies.

Further insights into infections are described by Mahroum et al. [9] who describe an increased prevalence of hepatitis C virus in SLE patients. Segal et al. [10] provide evidence for a possible role of human papilloma virus in SLE and support Shoenfeld’s concept of SLE as an autoimmune mosaic disease.

It is well recognised that B cell malignancies are over-represented in patients with SLE. In a large study from Israel, Azrielant et al. [11] utilised data from a health service database and confirmed the increased risk of non-Hodgkin’s lymphoma, Hodgkin’s lymphoma, multiple myeloma and other malignancies. This data emphasises the importance of screening patients with SLE for haematological malignancies in this patient group.

Antiphospholipid syndrome increases the risk of premature mortality and contributes significantly to morbidity in patients with SLE; therefore, the final section of this issue is devoted to this autoimmune thrombotic disorder. The classical autoantibodies associated with this syndrome are the lupus anticoagulant, anticardiolipin and anti-β2 glycoprotein 1 antibodies. Alessandri et al. [12] describe exciting data on the potential use of antibodies to mutated citrullinated vimentin (MCV) as a marker for the antiphospholipid syndrome. They found a significant correlation between anti-MCV and anti-vimentin/cardiolipin antibodies serum levels, and this data offer useful insights into alternative tests for the diagnosis of APS.

Stroke is a feared complication of APS, and Carmel-Neiderman et al. [13] assessed the value of classical and alternative autoantibody profiles in patients with strokes and carotid disease. They suggest that lower cut-off values of these autoantibodies than those used for APS diagnosis could be useful for risk stratification of CVA among healthy individuals, but this clearly needs further validation in what is a very controversial area.

Finally, the early detection of subclinical vascular disease may be a powerful tool to predict future cardiovascular events. Saponjski et al. [14] used 64-multi-sliced computed tomography to analyse lower limb vascular lesions and found a high prevalence of vascular lesions in patients with APS. They argue that this technique may be more accurate than ultrasound, but the downside is the use of radiation.

This issue offers a compelling collection of incisive articles for the reader to get up to date with advances in the fields of SLE and APS, and we congratulate the many authors on their timely contributions.
